# Modern Methods of Treatment of Simple Fractures

**Published:** 1894-02-17

**Authors:** 


### MODERN METHODS OF TREATMENT OF SIMPLE FRACTURES.

In our last issue we discussed some of the more striking improvements that have been made in the treatment of compound fractures within the last twenty or thirty years. We pointed out that to these improvements is to be traced an enormous saving of life and of limb. In this article, which deals with the treatment of simple fractures, we have no such striking results to chronicle. Life is rarely endangered by such injuries, and a limb is not often so gravely injured by a simple fracture that the question of amputation can be raised. Nevertheless, simple fractures are often very serious injuries, their management taxes the resources of the surgeon to the full, and their effects may be permanently disabling. They are generally looked upon as uninteresting and common-place "cases," and are liable to be a little neglected. But as the result of patient observation and a good deal of thought, something has been done of late years to make this branch of surgical practice more efficient, and it may be interesting to our readers to recall, at any rate, some of the improvements which have been introduced.

In the first place, we now have more exact diagnosis. In the case of fractures into or near a joint, especially the elbow joint, the fact of a fracture was held to be sufficient in the way of diagnosis, and precision in the recognition of the exact line of fracture was not considered important. We now know that exact diagnosis of the lesion is all-important, for it affords valuable help in prognosis, and enables the surgeon to proceed with the treatment with confidence. It is not enough to be able to recognise that there is a fracture, and then to indulge the good hope that as it is a "simple" one all must go well. Under such circumstances all is very likely to go wrong. The line of fracture not being determined, the fragments are not properly replaced, and union takes place with a deformed bone, and oftentimes a more or less stiff joint. At nearly every examination in clinical surgery in London cases of old simple fracture about the elbow or ankle, with serious impairment of function, are shown, indicating how common a result this is. Where the usual examination fails to clear up the diagnosis, chloroform should be given, and under the muscular relaxation it induces a further examination should be made unfettered by signs of pain. When the present writer was a student he was taught that it was a dangerous and serious error to give an anæsthetic for a simple fracture, that the sharp fragments of bone were liable to be thrust through the muscles and skin during the struggles of the patient, and that no corresponding advantage was to be obtained. We now know better. Anæsthesia is a help, and only a help where properly employed, and many fractures can only be properly treated with its aid.

The next great improvement has been the full recognition of the fact that perfect success in the management of fractures is only attainable when the broken fragments are perfectly restored to their proper position. Partial reduction was a frequent cause of disaster. It was hoped that Nature would remedy any defects in the "setting" of a fracture, but the hopes were always blighted; and it has been shown that no care in correcting displacement in a fracture is excessive. Here, again, chloroform, by relaxing all muscles, and preventing any voluntary or involuntary movements on the part of the patient, is of the utmost service. The fact is that the primary dressing of a simple fracture is a delicate and precise operation, and should be undertaken with all the care and under the same advantages as any other delicate surgical operation is. No one would think of operating for cataract in a crying, struggling patient; and in the same way we should not spoil our fracture results by having the "setting" marred by struggles, spasms, and cries.

A third very great improvement that has been effected is in the splints and retentive apparatus that is used. Surgeons have learnt the lesson that Mr. Bright essayed to teach politicians, that "force is no remedy." We no longer pull and push, drag or press, our broken limbs into position. But having corrected displacement completely, well-fitting splints are supplied with moderate equable pressure, and the parts held in place. We have learnt that the restraining power of slight but equable and general pressure is vastly greater than that of greater force badly applied. Moulded splints, accurately fitting the limb, are largely replacing flat wooden splints, and when it is more widely realised that the best moulded splint is made out of coarse house flannel cut to size and soaked in plaster of Paris—materials always at hand—we shall see wooden splints still further discarded.

More care is taken now than formerly to restore the function of muscles and joints which have been impaired by the orginal injury, and by the treatment to which it gave rise. It is recognised as an integral part of treatment to send the patient away, not only with a united bone, but a useful limb, strong to support, quick and secure in movement. The cases of bonesetters have been largely made up of those who have been more or less neglected in this particular. The most recent suggestion on this point is to remove the splints from the limbs in about ten days, and from that time only apply massage vigorously to the muscles and joints of the injured member. It is claimed for this treatment that it expedites the consolidation of the fracture and the removal of the excess of callus—"provisional callus," and restores the full function of the part in little more than half the time generally required. This treatment has obtained some support in Germany and Austria, but has not been very favourably received here. Nevertheless, it is a treatment which has its merits, and will be of special value where in addition to a broken bone the muscles and joints are contused, torn, or strained.

To sum up them, the modern methods of treating simple fractures are improvements upon the former, inasmuch as they first of all start from a more exact diagnosis of the injury, aim at a complete "reduction" of the fragments, maintained not by force, but by well-fitting retentive splints, and followed up by due attention to the condition of the joints, muscles, vessels, and nerves of the injured part.

